# Monochorionic diamniotic twins with centrally located and closely spaced umbilical cord insertions in the placenta

**DOI:** 10.1002/ccr3.1332

**Published:** 2018-01-03

**Authors:** Takashi Imamura, Hajime Maeda, Hidetoshi Kinoshita, Shogo Kin

**Affiliations:** ^1^ Department of Pediatrics Takeda General Hospital Aizu Wakamatsu Fukushima 965‐8585 Japan; ^2^ Department of Obstetrics and Gynecology Takeda General Hospital Aizu Wakamatsu Fukushima 965‐8585 Japan

**Keywords:** Central umbilical cord insertion, monochorionic diamniotic placenta, small intercord distance, vascular anastomoses

## Abstract

When to deliver the monochorionic diamniotic (MCDA) twins with specific cord patterns? Although there is no clear evidence supporting an earlier delivery (before 36 weeks of gestation) in MCDA twins, an earlier delivery might prevent intrauterine death or neuromorbidity in MCDA twins with specific cord patterns.

## Introduction

The condition in which twins share a single gestational sac is defined as a monochorionic pregnancy. The presence or absence of the amnion is determined after 7 weeks of gestation 1. A monochorionic diamniotic (MCDA) placenta is defined as a double amniotic cavity with a single placenta and both umbilical cord insertions (UCIs) in each cavity. In MCDA twins, vascular anastomoses are nearly always present and are thought to be responsible for the development of complications in pregnancy, such as twin‐to‐twin transfusion syndrome (TTTS) and damage to the surviving twin in the event of the intrauterine death (IUD) of its cotwin 2, 3. The cords are most commonly located at a distance from each other, unlike that observed in the monochorionic monoamniotic (MCMA) twin placenta. However, here, we present a rare type of MCDA placenta with two UCIs that were located centrally and in close proximity, such as observed in the MCMA twin placenta.

## Case Report

**Figure 1:**
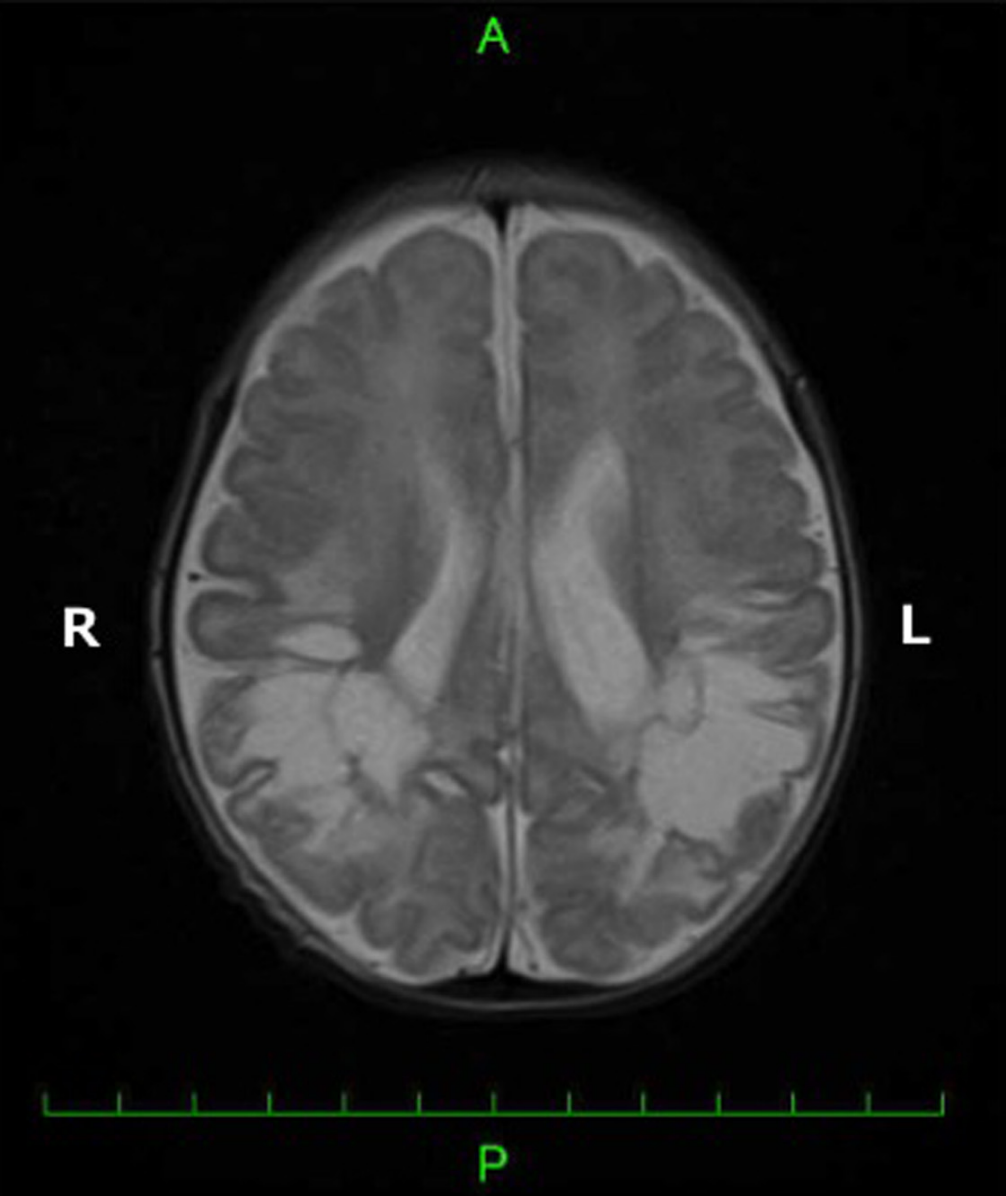
Large periventricular leukomalacia diagnosed on the basis of magnetic resonance imaging findings (T2‐weighted imaging sequences in the transverse plane).

**Figure 2:**
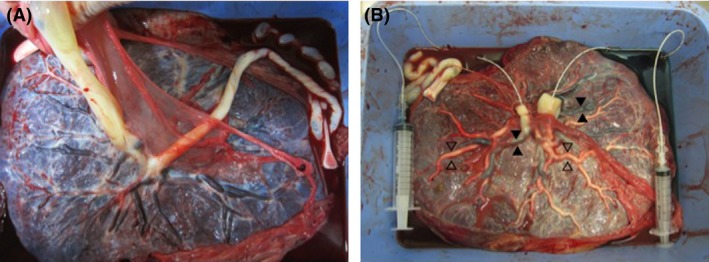
(A) An monochorionic diamniotic (MCDA) placenta with central cord insertions and a small intercord distance. The surviving infant's cord was thick (left) while that of the dead fetus was thin (right). (B) An MCDA placenta with central cord insertions and a small intercord distance is shown after injection testing using milk and indigotindisulfonate sodium. The milk and indigotindisulfonate sodium were injected into the umbilical vein of the surviving infant (thick cord) and umbilical arteries of the dead infant (thin cord), respectively (veins are white and arteries are sky blue). Several dynamic superficial venovenous (white arrow) and arterio‐arterial (black arrow) anastomoses can be seen in the whole placenta.

A 34‐year‐old Japanese woman in her second spontaneous pregnancy was referred to Takeda General Hospital at 17 weeks of gestation and diagnosed with a MCDA twin pregnancy. She was admitted to our hospital at 30 weeks' for management of potential premature delivery. She was regularly monitored by conventional ultrasound to assess growth and amniotic fluid volume, and by Doppler ultrasound of the umbilical artery (Table 1). No TTTS complications were observed during hospitalization. The final routine monitoring before delivery was performed at 35 weeks and 5 days of gestation; and the maximum vertical pockets of the MCDA twins were observed to be 4.2 and 3.6 cm, respectively, with cardiotocography showing reassuring fetal status patterns for both. However, she complained of diminished fetal movement at 35 weeks and 6 days of gestation (approximately 12 h later; at the final confirmation of normal cardiac sound for both twins by fetal Doppler ultrasonography), and the IUD of one fetus was confirmed by ultrasonography. Emergency cesarean section was performed, and the patient delivered a 2306 g surviving twin male infant, and a 1994 g dead twin male infant without any definite anomalies. No autopsy was performed as consent could not be obtained from the parents. The surviving infant's hemoglobin was 13.9 g/dL, and ultrasonography of the head revealed no abnormal findings at birth. Although he showed no cardiac or renal dysfunction after birth, he was diagnosed with large cystic periventricular leukomalacia (PVL) on the basis of magnetic resonance imaging findings at 13 days after birth (Fig. 1). His placenta was peculiar in that both UCIs were observed to be centrally located and in close proximity on the placenta (Fig. 2A). We did not observe any specific placental and umbilical cord findings during the fetal period. The placenta was 24 × 19 cm and weighed 778 g. The umbilical cords were found to be of unusual thickness and of 45 and 48 cm in length, respectively. Both umbilical cords were composed of double arteries and a single vein, with neither wrapped around the fetus's neck. There was no overcoiling or undercoiling of the umbilical cord vessels. After delivery, placental injection studies using milk and indigotindisulfonate sodium were performed. The vein and arteries of both umbilical cords were cannulated successively with a 3.5‐mm umbilical catheter, and milk and indigotindisulfonate sodium were injected into the umbilical vein of the surviving infant and umbilical arteries of the dead infant, respectively. The presence of several dynamic superficial venovenous (VV) and arterio‐arterial (AA) anastomoses was confirmed (Fig. 2B). A cross‐section of the placenta showed no calcification, hematoma, or infarction.

**Table 1:** Ultrasonographic study of monochorionic diamniotic twins during the third trimester

Gestational age	Measurements	Surviving infant	Dead infant
28 weeks and 2 days	EFW, g (SD)	1118 (−0.7)	1081 (−1.3)
32 weeks and 2 days	EFW, g (SD)	1685 (−0.3)	1409 (−0.9)
	MVP, cm	5.4	5.3
33 weeks and 2 days	EFW, g (SD)	1884 (−0.6)	1601 (−1.8)
	MVP, cm	4.2	3.9
34 weeks and 2 days	EFW, g (SD)	2077 (−0.5)	1604 (−2.3)
	MVP, cm	4.3	4.2
	UmA RI	0.62	0.63
	MCA RI	Not measured	0.78
34 weeks and 5 days	EFW, g (SD)	2033 (−0.9)	1621 (−2.5)
	MVP, cm	4.8	4.2
	UmA RI	0.64	0.62
	MCA RI	0.82	0.8
35 weeks and 5 days	EFW, g (SD)	2214 (−0.9)	1780 (−2.4)
	MVP, cm	4.2	3.6
	UmA RI	0.64	0.48
	MCA RI	0.82	0.9

EFW, estimated fetal weight; SD, standard deviation; MVP, maximum vertical pocket; cm, centimeters; UmA RI, umbilical artery resistance index; MCA RI, middle cerebral artery resistance index.

## Discussion

This was our first experience of a MCDA placenta with both UCIs located centrally and in close proximity, as in a MCMA twin placenta. In this case, placental injection studies revealed the presence of several dynamic superficial VV and AA anastomoses. These factors may have resulted in the development of large cystic PVL in the surviving infant at 13 days after birth. It is likely that fetal blood flow through the intertwined vascular anastomoses is dynamically variable, and that the net result of the combination of anastomosis type could well be quite unpredictable. Lewi 3 reported that multiple vascular anastomoses may indeed cause a transitory cardiovascular imbalance that is severe enough to decrease brain perfusion and cause cerebral lesions without resulting in an IUD or clinically evident TTTS. Moreover, Hillman et al. 4, in a systematic review and meta‐analysis of the effects on the surviving twin of single fetal death, reported that monochorionic twins were 4.8 times more likely to experience neurodevelopmental morbidity. It is speculated that the transfusional effects associated with single fetal death in monochorionic twins are associated with transient hemodynamic fluctuations leading to a predisposition to ischemic white matter changes 5.

The number of anastomoses in a monochorionic twin placenta has been reported to be correlated with the distance separating the cord insertion sites 6. Kellow and Feldstein 7 suggested that monochorionic twins face unique potential complications related to their two cord insertions, such as a higher incidence of velamentous insertions and the intertwining of vascular connections in the single shared placenta. On the other hand, Hack et al. 8 reported that no associations existed between mortality and anastomosis type, or between type and distance between the UCIs or placental sharing in monoamniotic twins. The distance between the cord insertions does not seem to have any particular association with the four types (AA shunt, VV shunt, parenchymal, and component) of anastomoses known to form 6. Furthermore, it was speculated that most monochorionic placentas had more than one type of anastomosis 6. In this case, we considered that VV and AA transfusions in placentas with two centrally located UCIs may improve the discordance in birthweight between the twins due to equal placental sharing. These vascular anastomoses have been used to explain the consequences to the surviving twin in cases of the IUD of its cotwin, even if the hemodynamics were balanced to that point 6.

Ultrasound examination, particularly after 8 weeks of gestation, can reliably determine chorionicity and amnionicity in the first trimester 1, significantly benefiting fetal risk assessment and subsequent management decisions. Kaneko et al. 9 suggested that the MCDA twin score, which is composed of five variables (discordancy in the amniotic fluid, discordancy in birthweight, abnormal cord insertion, hydrops fetalis and abnormal fetal heart rate monitoring), had a higher likelihood ratio for predicting poor outcomes higher than did any single variable or combination of the five variables. The consequence of increased surveillance and referral for TTTS improved outcomes in MCDA pregnancies 10. However, to the best of our knowledge, there have been no reports describing this in detail for MCDA placentas with both UCIs located centrally and in close proximity throughout the gestational period. Thus, the importance of these findings remains controversial. If specific cord patterns, as in our case, are observed during early pregnancy, more careful and regularly evaluation by Doppler ultrasound study may be recommended.

Finally, our experiences give rise to the questions as to when to deliver MCDA twins with specific cord patterns (velamentous or central). Although Hack et al. 7suggested that delivery of MCDA twins with a single placenta and vascular anastomoses should be planned at 36 weeks of gestation. Cheong‐See et al. 11 also reported that there is insufficient evidence to recommended routine delivery before 36 weeks of gestation in MCDA twins. Although there is no clear evidence supporting an earlier delivery (before 36 weeks of gestation) in MCDA twins, an earlier delivery might prevent an IUD or neuromorbidity in MCDA twins with specific cord patterns. Clinical expectations can only be formulated as more cases are encountered, making further study essential to the development of effective management and strategies for MCDA twins with specific cord patterns during the perinatal period.

## Consent

Written informed consent to report this case study was obtained from the parents of this infant.

## Conflict of interest

None declared.

## Authorship

TI: drafted the first version of the manuscript, participated in clinical study design and interpretation, and read and approved the final manuscript. HM, HK, and SK: reviewed the manuscript critically, participated in clinical study design and interpretation, and read and approved the final manuscript.
